# Facile Preparation of Glass Fiber Wool/MTMS Aerogels with Improved Thermal Insulation and Safety

**DOI:** 10.3390/gels11110906

**Published:** 2025-11-12

**Authors:** Yong Ren, Huanlin Zhang, Xingwei Jiang, Miao Liu, Zhi Li

**Affiliations:** 1 China Communications Construction Co., Ltd., Beijing 100011, China; 2 China First Highway Engineering Co., Ltd., Suzhou 215011, China; 3 CCCC Construction Group Co., Ltd., Beijing 101304, China; 4 School of Resources and Safety Engineering, Central South University, Changsha 410083, China

**Keywords:** glass fiber wool, methyltrimethoxysilane aerogel, thermal insulation, hydrophobic properties, thermal safety performance

## Abstract

With the continuous increase in global energy consumption and the escalating severity of climate change, the development of high-performance thermal insulation materials is crucial for reducing energy waste and carbon emissions. In this work, a facile method was proposed to prepare thermal-insulating glass fiber wool/methyltrimethoxysilane aerogel (GFWA) composites through vacuum-assisted impregnation. The obtained results indicated that GFWA composites exhibited excellent thermal insulation and hydrophobic properties, with GFWA-30 containing 30 wt.% glass fiber wool having a thermal conductivity of 35.3 mW/m·K and a water contact angle of 125.8°. Additionally, the Young’s modulus of this composite was 21.2% higher than that of MTMS aerogel. In terms of thermal safety performance, compared to methyltrimethoxysilane aerogel, the GFWA-30 composite showed reductions of 21.6%, 18.8%, and 27.95% in peak heat release rate, total heat release, and gross calorific value, respectively. This study offers a simple and feasible approach to fabricating high-performance thermal insulation materials, which display huge potential for widespread application in the fields of building insulation and other fields with thermal insulation requirements.

## 1. Introduction

With the continuous increase in global energy demand and the growing need for energy conservation and environmental protection, there is an urgent need to develop high-performance thermal insulation materials to reduce energy consumption and carbon emissions, particularly in sectors such as building construction, industrial equipment, and transportation [1,2,3]. Silica aerogel (SA), with its unique nanoporous structure and exceptionally high porosity, is considered one of the most effective thermal insulation materials available today. Its thermal conductivity can be as low as 0.012–0.020 W/m·K, which is significantly lower than that of traditional insulation materials [4,5,6,7,8,9]. The unique structural features of SA effectively hinder the conduction and convection of gas molecules, while the solid skeleton, composed of nanoscale silica-oxygen networks, experiences a significant reduction in continuity, leading to disrupted heat transfer pathways that further decrease thermal conductivity [10,11,12]. Additionally, SA exhibits excellent thermal stability, lightweight properties, and environmental friendliness [13,14,15]. Therefore, SA is regarded as a highly promising thermal insulation material with considerable potential to significantly reduce energy consumption.

Among them, SA prepared with methyltrimethoxysilane (MTMS) as the precursor offer significant advantages in terms of synthesis compared to those prepared using tetraethyl orthosilicate (TEOS) or water glass [16,17,18]. On the one hand, MTMS-based silica aerogels (MSA) can be rapidly synthesized in a pure water system, reducing the environmental hazards and cost associated with organic solvents. Additionally, the gel can be dried under ambient pressure to form a complete structure, greatly improving the convenience of preparation and reducing dependence on experimental equipment. For instance, Huang et al. proposed a method for the rapid synthesis of MSA in pure water, which eliminates the need for solvent exchange, surface modification, and aging processes, allowing the MSA to be synthesized in pure water within 4 h under vigorous ambient pressure drying conditions [19]. Furthermore, the self-carrying methyl group in MTMS imparts inherent hydrophobicity to MSA, thus eliminating the need for surface modification or solvent exchange [20]. However, MSA still has inherent drawbacks, particularly its brittleness. The nanoporous structure of the aerogel inherently makes it a brittle material, limiting its ability to withstand mechanical loads under complex working conditions [21]. Additionally, the flammability of MSA remains a significant concern. The methyl groups present in MSA are flammable, limiting its use in applications that require fire resistance [22]. Wu et al. confirmed through cone calorimeter tests that MSA ignites under an external heat flux of 35–45 kW/m^2^, with the increase in heat flux accelerating combustion and degradation. The flammability is primarily attributed to the methyl groups in the silica network, which undergo thermal degradation at 300–400 °C, releasing flammable volatile compounds such as CH_4_ and CH_3_OH [23]. Therefore, improving the flame-retardant and mechanical properties of MSA is crucial.

To overcome the inherent limitations of MSA, researchers have employed various modification strategies, among which composite reinforcement is considered an effective approach. By combining MSA with fibers exhibiting excellent mechanical properties or flame-retardancy, the brittleness and flammability of MSA can be effectively improved. For example, Jiang et al. synthesized a novel room-temperature-dried MSA nanocomposite enhanced by smaller-diameter micron glass fiber mats, which exhibited a low thermal conductivity of 0.022 W/m·K and high mechanical performance, with a bending strength of 1.4 MPa [24]. Zhou et al. prepared glass fiber-reinforced MSA composites using MTMS and water glass as co-precursors via freeze-drying. Compared to pure SA, the composites demonstrated significant improvements in mechanical strength and flexibility; when the molar ratio of MTMS to water glass was 1.8, the composites showed a high specific surface area (870.9 m^2^/g), contact angle (150°), thermal stability (560 °C), and low thermal conductivity (0.025 W/m·K) [25]. Duan et al. successfully fabricated glass fiber-reinforced SA composites by dispersing SA particles in a PVA aqueous solution. These composites exhibited excellent mechanical strength, low thermal conductivity (0.036 W/m·K), low density (0.131 g/cm^3^), and outstanding flame retardancy, with a heat release rate reduced to 146.48 kW/m^2^ [26]. Liu et al. developed a theoretical framework to investigate the effective thermal conductivity of SA composites reinforced with hollow fibers at high temperatures. Their results showed that increasing the hollowness of doped fibers improved extinction efficiency in the short wavelength range (<5 μm). Additionally, hollow fibers with larger outer diameters, in addition to smaller-diameter fibers (<3 μm), effectively suppressed radiative heat transfer within SA at high temperatures [27]. These studies provide valuable theoretical guidance for designing and fabricating MSA composites with improved mechanical and flame-retardant properties.

Glass fiber wool is an inorganic fiber material made from molten glass fibers through a centrifugal process, characterized by good flexibility, high-temperature resistance, and non-flammability, which makes it widely used in thermal insulation and soundproofing applications [28,29]. Due to its low thermal conductivity, outstanding mechanical properties, and flame-retardant characteristics, glass fiber wool is considered a promising reinforcing material to improve the brittleness and flammability of MSA, thereby enhancing its overall performance. Therefore, the incorporation of glass fiber wool is expected to strengthen the mechanical properties of MSA, reduce the fire risk of the composite, and improve its thermal safety.

This study aims to leverage the advantages of a pure water system and ambient pressure drying process to prepare MSA. Using vacuum-assisted impregnation, glass fiber wool/MSA (GFWA) composites were successfully fabricated. The microstructure, mechanical, thermal insulation, and thermal safety performance of the GFWA composites were systematically investigated, and the enhancement effects of glass fiber wool was studied. This study provides a novel solution for improving the inherent brittleness and flammability of MSA and lays the foundation for its broader application, particularly in building insulation, transportation, and industrial thermal insulation, demonstrating significant potential for future applications.

## 2. Results and Discussion

### 2.1. Microstructure

**Figure 1:**
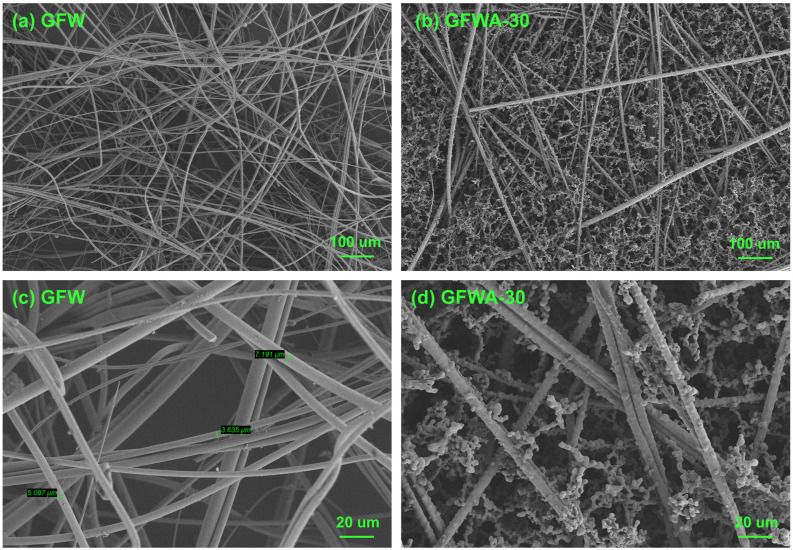
Microstructures of the GFW and GFWA-30. (**a**) SEM image of GFW at 100 magnification. (**b**) SEM image of GFWA-30 at 100 magnification. (**c**) SEM image of GFW at 500 magnification. (**d**) SEM image of GFWA-30 at 500 magnification.

The MTMS-derived aerogel is referred to as MTMS aerogel. The composites of aerogel doped with different amounts of GFW are referred to as GFWA composites. Specifically, the composites doped with 15 wt.%, 20 wt.%, 25 wt.%, and 30 wt.% of GFW are designated as GFWA-15, GFWA-20, GFWA-25, and GFWA-30, respectively. The microstructures of GFW and GFWA are shown in Figure 1. Pure GFW exhibits a state where a large number of fibers are intertwined randomly. The fibers are relatively dispersed, and the overall structure is loose, with obvious gaps between the fibers. In addition, there are certain differences in the diameters of the glass fibers, which range from 3 to 10 μm. The surfaces of the fibers are relatively smooth without obvious attachments, and the fibers are mainly simply physically intertwined [30]. In the microstructure of the composite material, the glass fibers still maintain a randomly intertwined state and are interspersed in the porous network structure of the MTMS aerogel. The porous network of the MTMS aerogel also fills the gaps between the fibers, making the overall structure denser. Notably, in Figure 1d, it can be observed that the surface of the glass fibers is no longer smooth, and a large number of MTMS aerogel particles are attached to the fiber surface. The MTMS aerogel has good contact with the fiber surface, and the interface bonding effect between them is excellent.

### 2.2. Surface Chemical Property and Hydrophobicity

**Figure 2:**
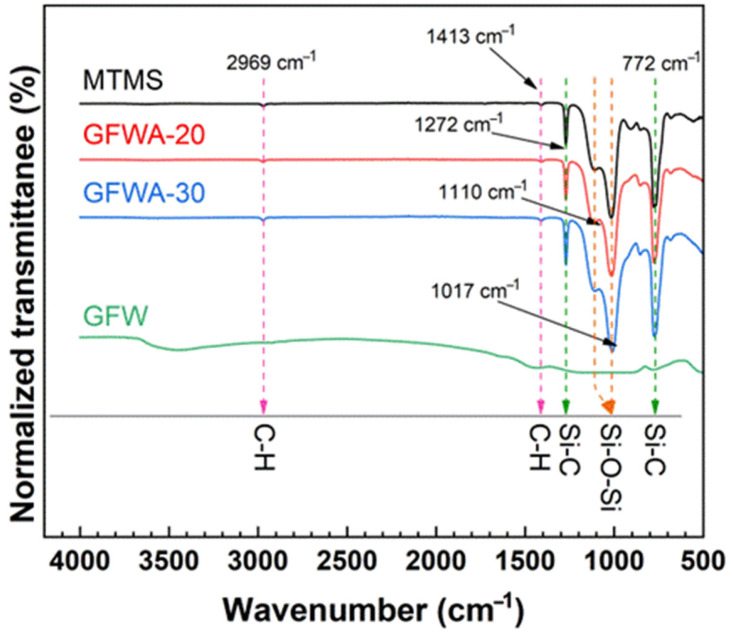
FTIR spectra of the MTMS aerogel, GFW and GFWA composites.

Figure 2 displays the FTIR spectra of MTMS aerogel, GFW, GFWA-20, and GFWA-30. With respect to MTMS aerogel, weak peaks were identified at 2969 cm^−1^ and 1413 cm^−1^, which correspond to the symmetric and asymmetric stretching vibrations of C-H bonds (-CH_3_ groups), respectively [31]. Further, the stretching vibration of Si-C bonds was detected at 1272 cm^−1^ and 772 cm^−1^. The peaks appearing at 1017 cm^−1^ and 1110 cm^−1^ were attributed to the asymmetric stretching vibration of Si-O-Si bonds, which served as an indication of a silica network [32]. Analysis revealed that the spectra of GFWA-20 and GFWA-30 were closely analogous to the superposition of the FTIR spectra of MTMS aerogel and GFW, and no newly formed absorption peaks were observed. This finding demonstrates that the interaction between MTMS aerogel and GFW was of a physical bonding nature. GFW was embedded into the pores of MTMS aerogel through physical interactions, and together they constitute a bulk aerogel composite with stable structural properties.

**Figure 3:**
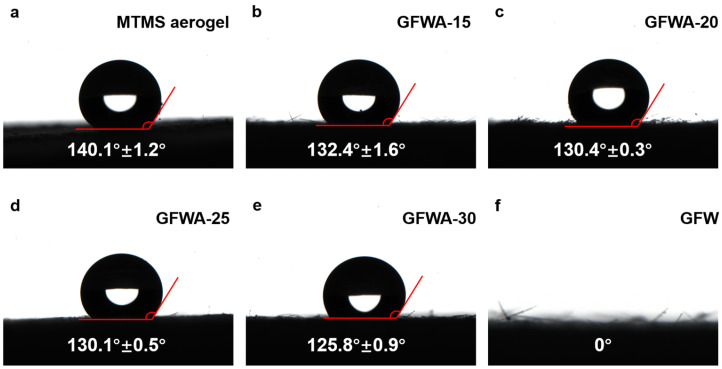
Contact angles of the MTMS aerogel (**a**), GFWA-15 (**b**), GFWA-20 (**c**), GFWA-25 (**d**), GFWA-30 (**e**), and GFW (**f**).

As shown in Figure 3, the contact angle of MTMS aerogel was 140.1°, demonstrating excellent hydrophobicity. However, when GFW was incorporated into the MTMS aerogel matrix, the hydrophobicity gradually decreased with increasing doping levels. The contact angle decreased from 132° for GFWA-15 to 125° for GFWA-30. This reduction in hydrophobicity was attributed to the hydrophilic nature of GFW, which showed a contact angle of 0°, indicating complete hydrophilicity. Therefore, the addition of GFW led to a slight weakening of the hydrophobicity of the composite. However, regardless of the GFW loading, the contact angle of the resulting GFWA composites remained above 125°, thus maintaining excellent hydrophobicity.

This phenomenon is attributed to the presence of Si-C and C-H bonds (confirmed by FTIR spectra), which form the chemical basis for the hydrophobicity of the composites. From a microscopic perspective, MTMS aerogel fills the gaps between GFW fibers and forms an adherent aerogel coating on the surface of GFW fibers, fully encapsulating the hydrophilic GFW fibers within the porous network of MTMS aerogel. In this case, both the outer surface of the composite and the interface in contact with water are entirely composed of the hydrophobic framework of MTMS aerogel, which ultimately endows the composite with excellent hydrophobicity.

### 2.3. Density and Porosity

**Figure 4:**
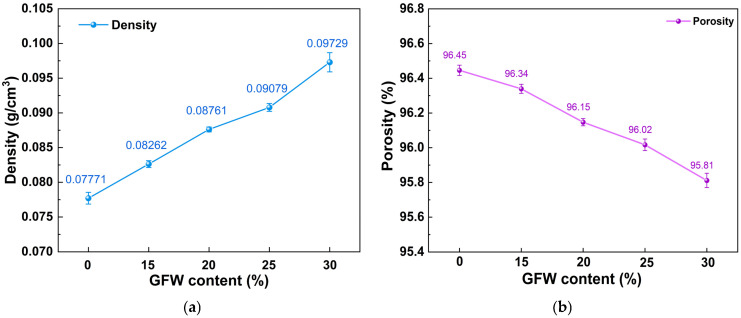
(**a**) Density and porosity (**b**) of MTMS aerogel and the GFWA composites.

Figure 4 presents the trends in density and porosity of GFWA composite aerogels with varying GFW contents. As shown in Figure 4a, the density of the composite increased significantly as the GFW content rose. When the GFW content was 0% (pure MTMS aerogel), the density was 0.07771 g/cm^3^. As the GFW content increased, the density of the GFWA composite rose to 0.09729 g/cm^3^ at 30 wt.% GFW. This increase in density was attributed to the relatively high skeletal density of GFW itself. As the GFW content increased, the proportion of the solid phase in the composite aerogel also increased, resulting in an overall increase in density.

In contrast to the density trend, the porosity of the GFWA composite aerogel decreased monotonically, as shown in Figure 4b. Specifically, the porosity decreased from 96.45% (0% GFW) to 95.81% (30 wt.% GFW). This decrease was due to the filling of the original pore space of the MTMS aerogel by GFW. As the GFW content increased, more fibers became embedded in the pore structure of the aerogel, which was consistent with the results observed via SEM. Additionally, during the impregnation of GFW into MTMS to form the composite aerogel, the GFW likely influenced the development of the aerogel’s network structure, further contributing to the reduction in porosity. Despite the reduction in porosity, even with 30 wt.% GFW, the composite aerogel maintained an ultra-high porosity (>95%), indicating that the addition of GFW did not significantly alter the aerogel’s structure.

### 2.4. Mechanical Property

**Figure 5:**
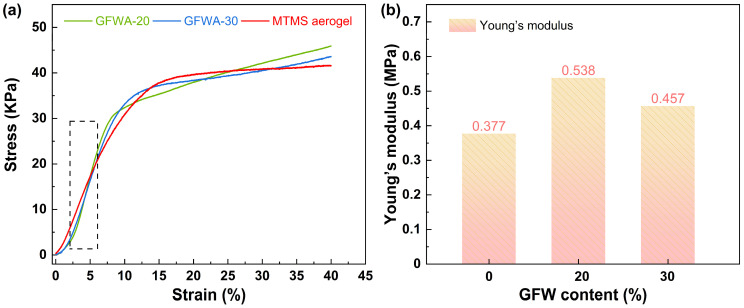
Compressive stress-strain curves (**a**), Young’s modulus and specific modulus (**b**) of the MTMS aerogel, GFWA-20 and GFWA-30. The region in the black dashed box between 2% and 6% is used to calculate the Young’s modulus.

Uniaxial compression tests were conducted on MTMS aerogel, GFWA-20, and GFWA-30 using a biomechanical testing machine, and the stress-strain curves are shown in Figure 5a. The stress-strain curves were divided into two stages. The first stage, the linear elastic stage (ε < 6%), exhibited a clear linear relationship between stress and strain, and the sample could completely recover its deformation after unloading the external force. Pure MTMS aerogel had a porous network structure. Within a certain stress range, the skeleton of the aerogel (a network composed of Si-O-Si bonds) absorbed energy through mechanisms such as bond stretching and bending, undergoing reversible elastic deformation. Glass fiber wool, which have high strength and elastic modulus, reinforced the composites when added to the MTMS aerogel. During the elastic stage, the glass fibers also bore part of the stress, enabling the composite to withstand greater external forces without permanent deformation. As shown in Figure 5b, the Young’s moduli of these composites with 20 wt.% and 30 wt.% GFW were 0.538 MPa and 0.457 MPa, respectively, both of which were higher than the 0.377 MPa of pure MTMS aerogel. Compared to pure MTMS aerogel, the Young’s moduli of GFWA-20 and GFWA-30 increased by 42.7% and 21.2%, respectively.

The second stage, the elastoplastic stage, corresponded to the range of 6% < ε < 40%, which was characterized as the plateau period. In this stage, stress increased slowly with strain, and both elastic and plastic deformations occurred simultaneously. After unloading the external force, irreversible deformation was observed. This was attributed to the collapse of some pores within the MTMS aerogel under the applied force.

### 2.5. Thermal Insulation Performance

**Figure 6:**
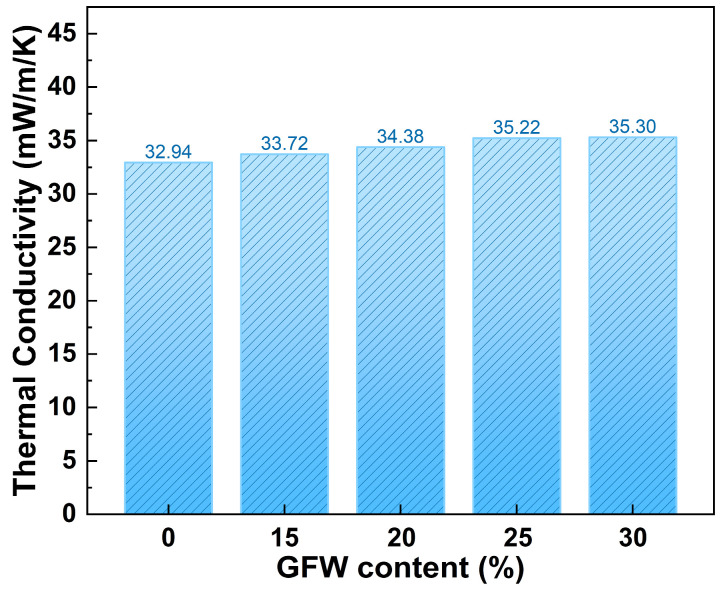
Thermal conductivity of MTMS aerogel and the GFWA composites.

The thermal conductivities of the MTMS aerogel and the GFWA composite aerogels are presented in Figure 6. It was observed that the thermal conductivity of the composite aerogels increased gradually with the rise in GFW content. Specifically, when the GFW content increased from 0 to 30 wt.%, the overall thermal conductivity rose from 32.94 mW/(m·K) to 35.30 mW/(m·K), corresponding to an increase of approximately 7.2%. The thermal conductivity of the material is influenced by the combined contributions of solid conduction, gaseous conduction, and radiative transfer. The pristine MTMS aerogel, due to its ultrahigh porosity, effectively suppressed gaseous conduction, resulting in an intrinsically low thermal conductivity. The incorporation of GFW partially occupied the pores within the MTMS aerogel, thereby enhancing solid-phase conduction. Additionally, as the GFW content increased, the porosity of the GFWA composite aerogels decreased, weakening the nanoscale pore structure’s ability to inhibit gaseous conduction. Notably, although thermal conductivity increased with higher GFW content, even at a 30 wt.% GFW loading, the composite aerogel still maintained a relatively low thermal conductivity. This indicated that the aerogel’s excellent thermal insulation was largely preserved, while achieving a favorable balance between mechanical reinforcement and thermal performance.

### 2.6. Thermal Safety

#### 2.6.1. Thermogravimetry-Differential Scanning Calorimetry Test

**Figure 7:**
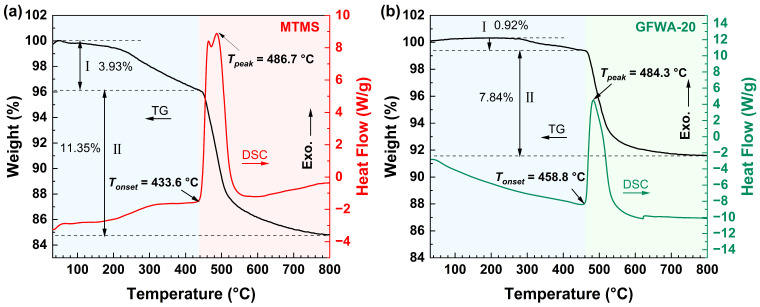
TG-DSC curves of the MTMS aerogel (**a**) and GFWA-20 (**b**).

As shown in Figure 7, thermogravimetric analysis was conducted on MTMS aerogel and GFWA-20 under an air atmosphere. The thermogravimetry (TG) curves of both materials exhibited a consistent trend, characterized by the weight loss process being divided into two distinct stages. Specifically, the initial stage showed a slight weight loss, which was attributed to the evaporation of residual water, adsorbed water, incompletely hydrolyzed precursors, and a small amount of CTAB in the framework. The mass loss of GFWA-20 in this stage was only 0.92%, much lower than the 3.93% observed for pure MTMS aerogel. This was because GFW interpenetrated and filled some pores of the MTMS aerogel, reducing the adsorption of physical water by the material. Additionally, the chemical inertness of glass fibers inhibited the volatilization of small-molecule organic components. As a result, the mass loss in the low-temperature section was significantly reduced, and the low-temperature thermal stability was greatly improved.

The main weight loss occurred in the second stage, which was attributed to the pyrolysis of Si-CH_3_, as evidenced by the obvious exothermic peak observed on the differential scanning calorimetry (DSC) curve. In this stage, the mass loss of GFWA-20 was only 7.84%, lower than the 11.35% observed for pure MTMS aerogel. This was due to the inorganic framework of GFW providing physical support for the organic phase of the MTMS aerogel, hindering the thermal decomposition path of organic components (by delaying oxygen diffusion and limiting the cracking rate of organic molecules). Therefore, the decomposition of the organic phase was inhibited, and the thermal stability of the composite was improved. GFWA-20 exhibited a higher residual mass, and the introduction of GFW enhanced the high-temperature residual capacity of the composite.

*T_onset_* and *T_peak_* represent the onset and peak temperatures of the thermal oxidative decomposition of Si-CH_3_, respectively, and can be used to evaluate the thermal stability of materials. From the DSC curves, the introduction of GFWA increased *T_onset_* from the original 433.6 °C to 458.8 °C, while *T_peak_* remained almost unchanged. This further indicates that due to the presence of GFW in the system, a physical barrier is provided for the organic phase of the MTMS aerogel, making the initiation of thermal decomposition of organic components more difficult and thus improving the thermal stability of the material.

#### 2.6.2. Gross Calorific Value

**Figure 8:**
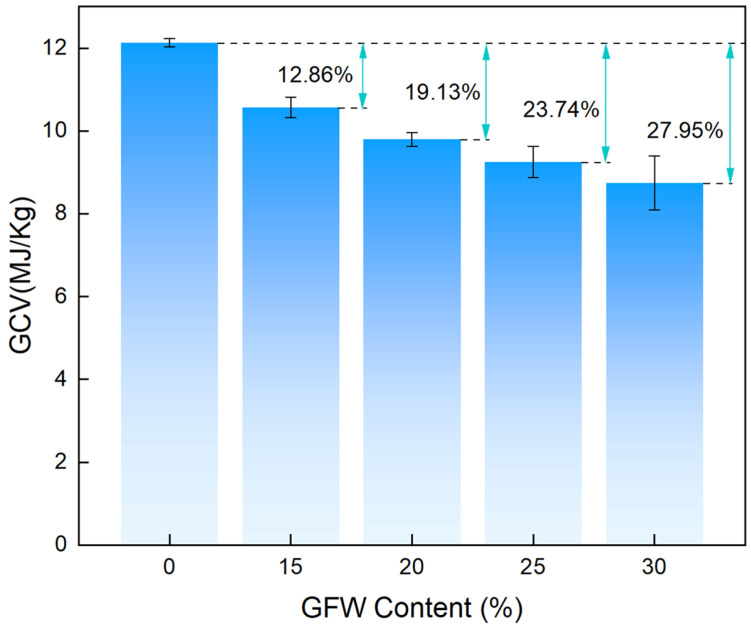
GCV of MTMS aerogel and the GFWA composites.

As shown in Figure 8, with the increase in GFW content (from 0 to 30 wt.%), the gross calorific value (GCV) of the GFWA composite exhibited a continuous decrease. When the GFW content was 0%, i.e., pure MTMS aerogel, the GCV was the highest, reaching 12.13 MJ/kg. As the GFW content increased to 15 wt.%, 20 wt.%, 25 wt.%, and 30 wt.%, the GCV decreased to 10.57 MJ/kg, 9.81 MJ/kg, 9.25 MJ/kg, and 8.74 MJ/kg, respectively, with reduction rates of 12.86%, 19.13%, 23.74%, and 27.95% compared to pure MTMS aerogel. GFW, an inorganic non-combustible material primarily composed of silicon dioxide, when incorporated into MTMS aerogel, diluted the content of combustible MTMS aerogel in the GFWA composite. As the GFW content increased, the relative proportion of combustible substances decreased, leading to a reduction in the total heat released during combustion. This finding provided an effective method for improving the flame-retardant performance of MTMS aerogel, which is of significant importance for expanding its application in fire-sensitive fields.

#### 2.6.3. Cone Calorimeter Test

**Figure 9:**
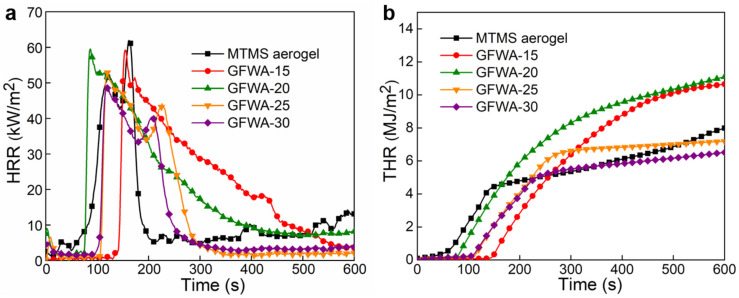
HRR (**a**) and THR (**b**) curves of the MTMS aerogel and GFWA composites.

Cone calorimeter test (CCT) evaluates the flame-retardant performance of materials by simulating real fire scenarios [33,34,35,36,37]. As shown in Figure 9, the pHRR and THR of MTMS aerogel were 61.96 kW/m^2^ and 8.0 MJ/m^2^, respectively. When GFW was incorporated, the pHRR of the GFWA composites showed an overall decreasing trend as the GFW content increases. Specifically, the GFWA-30 composite, containing 30 wt.% GFW, exhibited the lowest pHRR (48.56 kW/m^2^) among all samples, representing a 21.6% reduction compared to that of MTMS aerogel. Meanwhile, the THR initially increased and then decreased as GFW content increases. For example, the THR values of these composites with 20 wt.% and 30 wt.% GFW are 7.2 MJ/m^2^ and 6.5 MJ/m^2^, respectively, showing reductions of 10.0% and 18.8% compared to that of MTMS aerogel. This phenomenon may be attributed to the fact that at lower doping levels, the GFW is insufficient to fully exert its flame-retardant effect. Furthermore, the addition of GFW increases the thermal conductivity of the GFWA composite, enhancing heat transfer and consequently increasing heat release. When the doping level exceeds 20 wt.%, GFW acts as a thermal insulation barrier, effectively isolating oxygen and heat sources, thus reducing heat transfer and inhibiting the combustion process of the aerogel. Therefore, at higher doping levels, GFW slows down the rate of heat transfer, effectively suppresses heat release, and leads to a reduction in the pHRR and THR.

**Figure 10:**
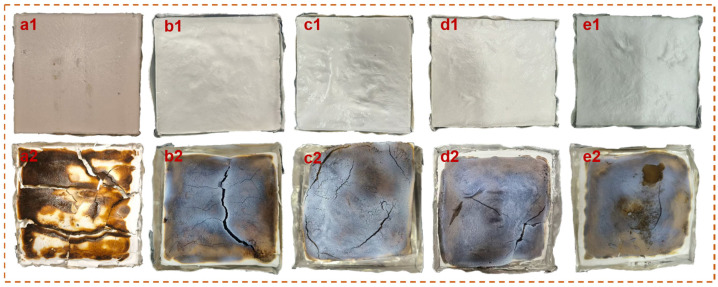
Digital photos of residual chars of the MTMS aerogel and GFWA composites before and after CCT: (**a1**,**a2**) MTMS aerogel, (**b1**,**b2**) GFWA-15, (**c1**,**c2**) GFWA-20, (**d1**,**d2**) GFWA-25, and (**e1**,**e2**) GFWA-30.

The photographs of MTMS aerogel and GFWA composites before and after CCT are shown in Figure 10. It was evident that the residual char in MTMS aerogel exhibited significant cracking and poor overall integrity. In contrast, the GFWA composites containing 15 wt.% or 20 wt.% GFW showed improved residual char integrity, although the surface remained relatively loose. As the GFW content increased, the extent of cracking in the GFWA composites decreased, and the integrity and density of the char layer gradually improved. In particular, the char layer of GFWA-30 displayed the best integrity and density among all the samples. The dense and intact char layer effectively blocks the transfer of heat and oxygen to the underlying unburned substrate, thereby effectively suppressing combustion. These findings suggest that the flame-retardant performance of the composites was enhanced.

### 2.7. Performance Comparison

To emphasize the superior performance of the synthesized GFWA composites, we compared them with previously reported thermal insulating aerogels, as shown in Table 1. Zhu et al. synthesized a flame-retardant thermal insulating aerogel (HANRs/SAB) using sodium alginate (SA) and hydroxyapatite nanorods (HANRs), which exhibited good flame-retardant properties and a low thermal conductivity of 40.9 mW/m·K [38]. Cui et al. prepared a phosphorylated chitosan aerogel (PCS), which demonstrated intrinsic flame retardancy and low thermal conductivity but lacked hydrophobicity [39]. Tao et al. synthesized a flame-retardant rigid polyurethane foam (RPUF/m-SA) for thermal insulation applications [40]. In addition to these works, several other researchers have explored thermal insulation aerogels, such as SiO_2_@carboxymethyl cellulose aerogel (SiO_2_@CMCA), MXene/carbonized bagasse fiber aerogel (MXene@CBF), and SiO_2_ aerogel/epoxy composites [41,42,43]. Although these materials have made some progress in thermal insulation performance, the GFWA composites we synthesized exhibit superior overall performance compared to the materials reported above.

## 3. Conclusions

In this study, we developed a facile preparation method for fabricating GFWA composites through vacuum-assisted impregnation of MTMS wet gel, followed by ambient pressure drying. The prepared GFWA composites exhibited excellent hydrophobicity, with contact angles greater than 125°. In terms of thermal insulation performance, although the doping of GFW results in a slight increase in thermal conductivity, the thermal conductivity of the composite with 30 wt.% GFW is only 35.30 mW/m·K. Regarding mechanical properties, the Young’s moduli of GFWA-30 increased by 21.2% compared to pure MTMS aerogel. Furthermore, the incorporation of GFW significantly improved the thermal safety performance of the GFWA composites. For instance, GFWA-30, which contains 30 wt.% GFW, demonstrated a 21.6% reduction in pHRR, a 18.8% reduction in THR, and a 27.95% reduction in GCV, compared to those of MTMS aerogel. This work provides a feasible and effective strategy for developing high-performance thermal insulation materials with excellent fire safety and hydrophobicity, and the as-created GFWA composites show considerable promise for a wide range of applications, particularly in energy-efficient building insulation, the petrochemical industry, and transportation.

## 4. Materials and Methods

### 4.1. Raw Materials

MTMS (98%) and cetyltrimethylammonium bromide (CTAB, 99%) were purchased from Aladdin Company (Shanghai, China) and used as the silicon source and surfactant, respectively. Glass fiber wool (GFW) was supplied by Deqing Guotai Fireproof Material Factory. Nitric acid (HNO_3_, 36–38%) and ammonia solution (NH_3_·H_2_O, 25–28%) were used as acid and alkali catalysts, respectively, and were purchased from Aladdin Company (Shanghai, China) and Sinopharm Chemical Reagent Co., Ltd. (Shanghai, China). Deionized water for experiments was prepared using an ultrapure water system (ECO-S, Hitech, Shanghai, China).

### 4.2. Preparation of GFW/MTMS Aerogel (GFWA) Composites

**Figure 11:**
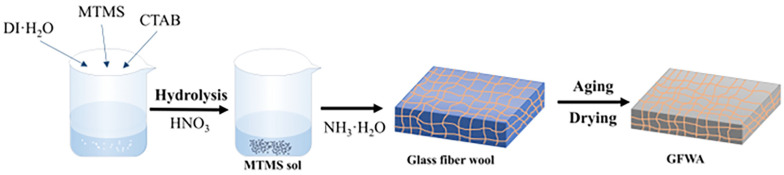
Schematic representation of the process to prepare the GFWA composites.

The preparation of the GFWA composites consists of two main steps: the preparation of the MTMS wet gel and the impregnation of GFW with the wet gel. The detailed process is outlined in Figure 11.

First, a specified mass of H_2_O, CTAB, and MTMS was mixed in a 100 mL beaker and stirred for several minutes. A certain volume of 0.1 M nitric acid (HNO_3_) was then added, and the mixture was placed in a 30 °C water bath for hydrolysis for a specific period. After hydrolysis, the MTMS hydrolyzate was transferred to a refrigerator to cool, and 1 M ammonia solution (NH_3_·H_2_O) was added to the cooled sol to obtain the MTMS wet gel for subsequent use.

Next, the GFW was cut to a size matching the silica gel mold (50 mm × 50 mm × 25 mm) and dried at 120 °C for 4 h to remove adsorbed water. The dried GFW was then laid flat at the bottom of the mold. Based on mass ratios of GFW constituting 0%, 15%, 20%, 25%, and 30% of the total mass of the system, corresponding volumes of MTMS wet gel were poured into the mold for vacuum impregnation. Finally, the samples labeled MTMS aerogel, GFWA-15, GFWA-20, GFWA-25, and GFWA-30 were obtained by atmospheric pressure drying.

### 4.3. Methods of Characterization

The microstructure of GFWA composite aerogels was characterized using a field emission scanning electron microscope (SEM, Zeiss Sigma 300, Oberkochen, Germany). The apparent density (*ρ*) was calculated by weighing GFWA bulk samples with regular geometric shapes. The porosity of the composites was determined using Equations (1) and (2).

(1) $$\mathrm{Porosity}=\left(1-\frac{\rho }{\rho_{s}}\right)\times 100\%$$

(2) $$\frac{1}{\rho_{s}}=\frac{c}{\rho_{GFW}}+\frac{1-c}{\rho_{SA}}$$

Among the parameters, *ρ_S_* denotes the skeletal density of the composite. In the experiment, the skeletal density of the glass fiber (*ρ_GFW_*) used was 2.6 g/cm^3^, while that of the silica aerogel (*ρ_SA_*) was 2.2 g/cm^3^. Here, c represents the mass fraction of GFW in the composite.

The mechanical properties of GFWA composites were investigated via uniaxial compression measurements with a loading rate set at 2 mm/min. Fourier transform infrared spectroscopy (FTIR, Thermo Nicolet iS50, Waltham, MA, USA) was employed to identify the chemical groups and bonds present in the composites, where the attenuated total reflection (ATR) method was used for sample preparation to ensure minimal sample manipulation and accurate data acquisition. Hydrophobicity was evaluated by the sessile drop method, and the contact angles were measured using an automatic instrument (JC2000D1, Zhongchen Instruments, Shanghai, China).

Thermal stability was evaluated via TG-DSC tests using a synchronous thermal analyzer (STA 8000, PerkinElmer, Shelton, CT, USA) under an air atmosphere. The heating rate was set at 10 °C/min, with the temperature ranging from room temperature to 800 °C. The GCV of GFWA composites was measured using an oxygen bomb calorimeter (AM-C1009, Yuanfa Instrument Co., Ltd., Changsha, China), aiming to quantify the energy released during the combustion process. The flame-retardant properties of all samples were evaluated using a cone calorimeter (TTechKZG06, TESTech Instrument Technologies Co., Ltd., Suzhou, China) under a heat flux of 35 kW/m^2^.

## Figures and Tables

**Table 1:** Comparison of flame retardancy, thermal conductivity, and water contact angle of thermal insulation aerogel materials.

Sample	Flame Retardancy	Thermal Conductivity (mW/m·K)	Water Contact Angle (°)	Ref.
HANRs/SAB	Yes	40.9	/	[38]
PCS	Yes	36–37	0	[39]
RPUF/m-SA	Yes	40.1	/	[40]
SiO_2_@CMCA	No	86	120.0	[41]
MXene/CBF aerogels	No	69	/	[42]
SiO_2_ aerogel/epoxy	No	90–100	124.0	[43]
GFWA-30	Yes	35.3	125.8	This work

## Data Availability

The original contributions presented in this study are included in the article. Further inquiries can be directed to the corresponding authors.
